# In Vitro Susceptibility and Molecular Docking of Echinocandins Against Clinical Dermatophyte Isolates: Implications for Resistant Dermatophytosis

**DOI:** 10.3390/jof12100769

**Published:** 2026-10-14

**Authors:** Iman Haghani, Iman Nikjan, Zahra Yahyazadeh, Milad Lagzian, Reza Valadan, Javad Javidnia, Tahereh Shokohi, Mohammad Taghi Hedayati, Jamshid Yazdani, Mohsen Nosratabadi, Narges Vaseghi, Mahdi Abastabar, Hamid Badali

**Affiliations:** 1 Invasive Fungi Research Center, Communicable Diseases Institute, Mazandaran University of Medical Sciences, Sari 48157-33971, Iran; imaan.haghani@gmail.com (I.H.); zahra_yahyazadeh77@yahoo.com (Z.Y.); javidniaj@gmail.com (J.J.); shokohi.tahereh@gmail.com (T.S.); hedayatimt@gmail.com (M.T.H.); 2 Department of Medical Mycology, School of Medicine, Mazandaran University of Medical Sciences, Sari 48157-33971, Iran; 3 Student Research Committee, Faculty of Medicine, Mazandaran University of Medical Sciences, Sari 48157-33971, Iran; imannikjan1@gmail.com; 4 Department of Biology, Faculty of Sciences, University of Sistan and Baluchestan, Zahedan 98167-45845, Iran; m.lagzian@science.usb.ac.ir; 5 Molecular and Cell Biology Research Center (MCBRC), Mazandaran University of Medical Sciences, Sari 48157-33971, Iran; valadan.reza@gmail.com; 6 Department of Biostatistics, Faculty of Health, Mazandaran University of Medical Sciences, Sari 48157-33971, Iran; jamshid_1380@yahoo.com; 7 Department of Laboratory Sciences, Sirjan School of Medical Sciences, Sirjan 76169-16338, Iran; nosratabadi.mohsen@yahoo.com; 8 Department of Pathobiology, Science and Research Branch, Islamic Azad University, Tehran 14778-93855, Iran; nvaseghi@yahoo.com; 9 Department of Molecular Microbiology & Immunology and South Texas Center for Emerging Infectious Diseases, The University of Texas at San Antonio, San Antonio, TX 78249, USA

**Keywords:** echinocandins, dermatophytes, *Trichophyton indotineae*, anidulafungin, caspofungin, resistant dermatophytosis

## Abstract

Drug-resistant dermatophytosis has emerged as a global therapeutic challenge, underscoring the need for alternative antifungal strategies. Echinocandins, inhibitors of β-(1,3)-D-glucan synthase, demonstrate potent activity against *Candida* and *Aspergillus* but remain insufficiently characterized against dermatophytes. This study evaluated the in vitro activity of anidulafungin and caspofungin in a diverse clinical collection and examined their predicted interactions with β-(1,3)-D-glucan synthase. Antifungal susceptibility testing was performed on 105 clinical isolates, including *T. indotineae*, *T. mentagrophytes*, *T. rubrum*, *T. tonsurans*, *M. canis*, *N. gypsea*, and *T. violaceum*, using CLSI M38-A3 broth microdilution. Minimum effective concentrations (MECs) for echinocandins and minimum inhibitory concentrations (MICs) for comparator agents were determined. Molecular docking simulations were conducted to characterize echinocandin interactions with β-(1,3)-D-glucan synthase. Statistical analyses used Kruskal–Wallis and Dunn’s tests. Both echinocandins showed significantly lower MEC/MIC distributions than all comparator antifungals after adjustment for multiple comparisons (all adjusted *p* < 0.001). Anidulafungin was more potent than caspofungin (GM MECs: 0.011 vs. 0.022 µg/mL; *p* < 0.001). Against terbinafine-resistant *T. indotineae*, MEC_50_ values were 0.008 µg/mL (anidulafungin) and 0.016 µg/mL (caspofungin), markedly outperforming terbinafine (MIC_50_ = 4 µg/mL). Docking analyses demonstrated species- and ligand-dependent predicted interaction patterns with β-(1,3)-D-glucan synthase, providing complementary structural context for the observed in vitro susceptibility profiles. This study extends prior in vitro observations by profiling two echinocandins against diverse clinical dermatophytes, including terbinafine-resistant *T. indotineae*. Docking analysis provides structural context but does not establish target engagement. Further preclinical studies are needed to confirm therapeutic relevance.

## 1. Introduction

Dermatophytosis accounts for 20–25% of all superficial fungal infections worldwide. It is caused by keratinophilic dermatophytes that secrete keratinase, enabling infection of the skin, hair, and nails [1]. Recent taxonomic revisions recognize 52 species distributed across nine genera—*Trichophyton*, *Microsporum*, *Epidermophyton*, *Arthroderma*, *Lophophyton*, *Nannizzia*, *Ctenomyces*, *Guarromyces*, and *Paraphyton* [1,2,3].

The significance of dermatophyte infections has dramatically increased globally, in both developed and developing regions. This rise is associated with widespread use of immunosuppressive medications and environmental factors such as hot, humid climates that favor fungal proliferation [4,5]. Although systemic itraconazole, terbinafine, griseofulvin, and fluconazole are currently effective antifungal agents [6], topical antifungal agents are considered the first-line treatment for uncomplicated superficial fungal skin infections, owing to their potent efficacy and minimal risk of systemic adverse effects. Research demonstrates that the success rates for clinical improvement and fungal eradication are similar across topical antifungal agents. However, drugs like terbinafine and butenafine are associated with longer-lasting remissions due to their fungicidal nature and residual skin activity [7].

However, a significant concern is the emergence of dermatophyte strains that are resistant to conventional treatments, especially terbinafine as a drug of choice, which has been identified as a public health challenge, causing patient despair [8]. These resistant strains were first reported in India in 2017 and were named *Trichophyton indotineae* and have rapidly spread to neighboring regions [9,10]. The prevalence of such cases has alarmingly increased, with at least 5 to 10 percent of new cases presenting to dermatology clinics being affected [11].

Given the high prevalence of dermatophytosis worldwide, as mentioned before, and the emerging challenges of resistant strains, the use of drugs with high efficacy and minimal adverse effects is recommended [12]. Despite their good efficacy, the azole compounds commonly used against dermatophytosis are linked to several side effects [13]. In cases involving resistant strains, itraconazole has become the preferred drug [9]. However, resistance to itraconazole has also been reported in some patients, further heightening concerns regarding treatment options [14].

Echinocandins inhibit β-(1,3)-D-glucan synthase, an essential fungal cell wall component [15]. In vitro studies have consistently shown low MECs of several echinocandins against dermatophytes, with caspofungin, anidulafungin, and micafungin demonstrating reproducible activity across multiple *Trichophyton* and *Epidermophyton* species and favorable activity against clinical *T. rubrum* isolates in recent investigations [16,17,18,19]. Importantly, Xie et al. reported favorable in vitro activity of echinocandins against 37 *T. indotineae* isolates, extending evidence of echinocandin activity to this clinically important dermatophyte [20]. Nevertheless, available studies have generally focused on selected dermatophyte species or species-specific collections, and systematic comparisons of echinocandin susceptibility across multiple clinical dermatophyte species, particularly within a resistance-enriched collection, remain relatively limited [16,17,18,19,20]. Importantly, based on previous research, the objective of the structural analysis was not to identify β-(1,3)-D-glucan synthase as a novel echinocandin target, which is well established, but to comparatively characterize the predicted interaction environments of anidulafungin and caspofungin across clinically relevant dermatophyte species. Recent experimental structural studies of fungal Fks1 have substantially advanced the understanding of echinocandin recognition and inhibition [21,22]. Nevertheless, species-resolved comparative structural analyses integrating predicted ligand-target interactions with experimentally determined susceptibility profiles across dermatophytes remain limited. Accordingly, the present docking analysis was designed as a complementary, hypothesis-generating component to provide structural context for the observed species- and ligand-dependent in vitro activity rather than as experimental confirmation of binding affinity or mechanism.

## 2. Materials and Methods

### 2.1. Sample Collection

Clinical isolates included *T. indotineae* (*n* = 20), *T. mentagrophytes* (*n* = 20), *T. tonsurans* (*n* = 20), *T. rubrum* (*n* = 10), *M. canis* (*n* = 20), *Nannizzia gypsea* (*n* = 10), and *T. violaceum* (*n* = 5) obtained from the Mazandaran University of Medical Sciences collection (Sari, Iran). All strains were previously identified using phenotypic, physiological, and molecular methods [23].

### 2.2. Antifungal Susceptibility Testing

Antifungal susceptibility testing was performed using broth microdilution methods based on the Clinical and Laboratory Standards Institute (CLSI) M38-A3 document [24], using RPMI 1640 medium (Sigma-Aldrich, St. Louis, MO, USA) buffered to pH 7.0 with 0.165 M 3-(N-morpholino) propanesulfonic acid (MOPS; Sigma-Aldrich, St. Louis, MO, USA), with L-glutamine and no bicarbonate [24]. Drug stock was further diluted 1/100 in standard RPMI 1640 medium. Final concentrations of 0.016–16 μg/mL for itraconazole (Janssen, Beerse, Belgium), voriconazole (Pfizer, Sandwich, UK), terbinafine (Sigma-Aldrich, St. Louis, MO, USA) and butenafine (Sigma-Aldrich, St. Louis, MO, USA), and 0.008–8 μg/mL for caspofungin (Merck Sharp and Dohme BV, Haarlem, The Netherlands) and anidulafungin (Pfizer, Central Research, UK) were used. Briefly, conidial suspensions were prepared by scraping the surface of mature colonies (5–10 days old) with a sterile cotton swab moistened with solutions prepared in dimethyl sulphoxide (DMSO, Sigma-Aldrich, St. Louis, MO, USA), except for echinocandins, which were dissolved in sterile water with a sterile physiological saline solution containing 0.05% Tween 20-40 and spectrophotometrically adjusted to optical densities (ODs) ranging from 65% to 70% transmission at a 530 nm wavelength. Inoculum suspensions were diluted 1:50 in RPMI 1640 medium, with the final inoculum in assay wells ranging from 1 to 3 × 10^3^ CFU/ml. The 96-well plates were incubated at 35 °C for 96 h. The minimum inhibitory concentration (MIC) was defined as the lowest concentration that completely inhibited fungal growth. In contrast, the minimum effective concentration (MEC) was determined microscopically; it is the lowest concentration of the drug that led to the development of small, round, compact hyphae, compared with the filamentous forms observed in the control wells. As quality control strains, *Candida parapsilosis* (ATCC 22019) and *Candida krusei* (ATCC 6258) were used, and all antifungal susceptibility tests were performed in duplicate [12]. As CLSI M38-A3 does not define clinical breakpoints or epidemiological cutoff values (ECVs) for dermatophytes tested against echinocandins, results were reported descriptively as MEC/MIC distributions, GM MECs, MEC_50_, and MEC_90_ values only, and no categorical interpretation was performed.

### 2.3. Three-Dimensional Structural Modeling via AlphaFold

The three-dimensional structures of β-(1,3)-D-glucan synthase across the designated fungal species were constructed using the AlphaFold3 server. The target amino acid sequences were supplied as queries to the AlphaFold pipeline, which leverages a deep learning-based neural network architecture to predict atomic coordinates with high accuracy. This computational methodology utilizes evolutionary information from multiple sequence alignments (MSAs) alongside structural information extracted from known protein templates in the Protein Data Bank (PDB). The resulting structural models were evaluated using the predicted local distance difference test (pLDDT) scores to confirm highly confident fold predictions across the core transmembrane and catalytic domains.

### 2.4. Binding Pocket Identification, Grid Generation, and Molecular Docking

**Figure 1:**
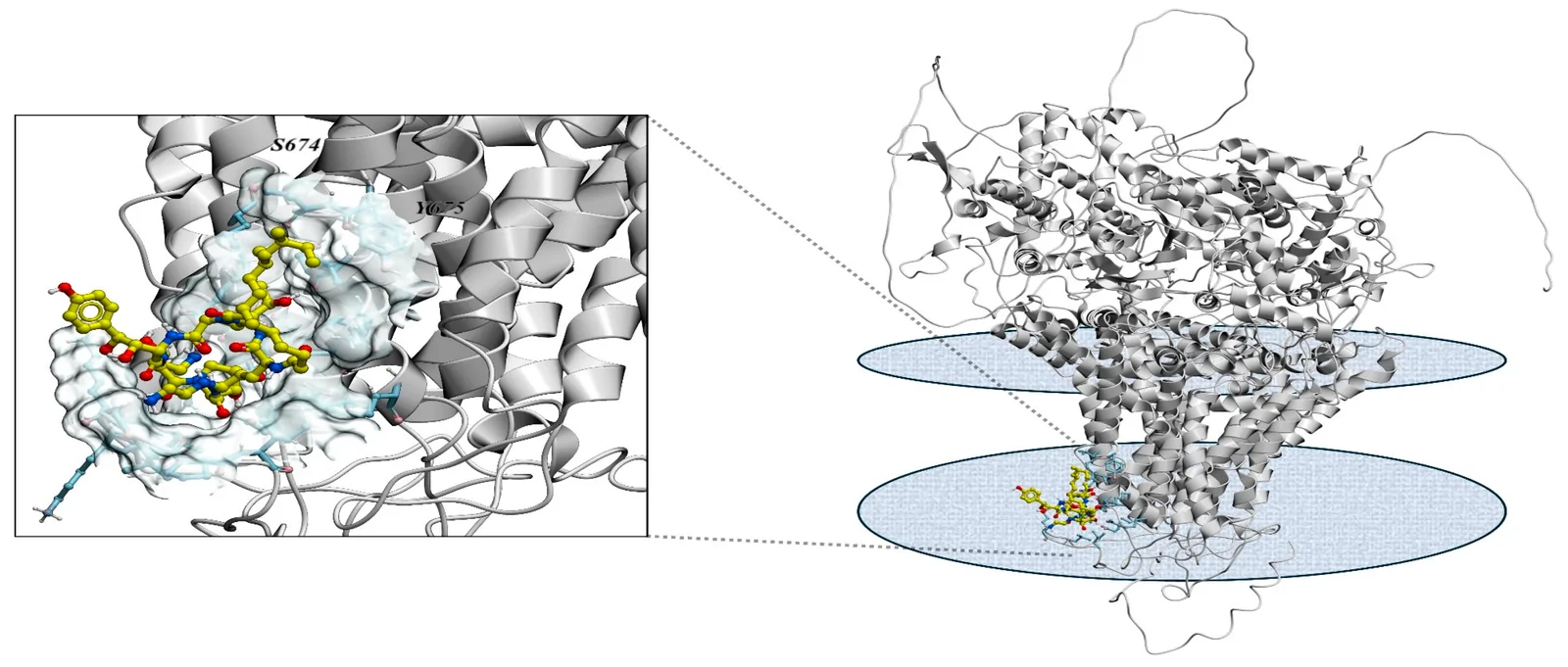
Molecular docking of caspofungin to *Trichophyton rubrum* β-(1,3)-D-glucan synthase. Representative example from the seven dermatophyte species analyzed in this study. (**Right**): Overall structure of the AlphaFold3-modeled enzyme with the protein shown in a gray/white ribbon representation and the transmembrane architecture illustrated within a schematically represented phospholipid bilayer (blue ellipses). The bilayer is shown for structural context only and was not explicitly modeled or simulated in the present computational analysis. (**Left**): Magnified view of the predicted caspofungin interaction region, highlighting the flexible residues Ser674 and Tyr675 used in the representative receptor grid setup. The drug’s hexapeptide core occupies the predicted interaction region, with a docking score of −6.005 kcal/mol. Atom colors in the ligand representation distinguish the different chemical elements.

To identify and map the precise echinocandin binding pocket on the modeled fungal proteins, a structural superimposition approach was utilized. The AlphaFold-generated structural models were aligned to the experimental reference structure of Saccharomyces cerevisiae β-(1,3)-D-glucan synthase captured in complex with caspofungin (PDB ID: 9PE1). Following the structural alignment of the backbone atoms, the coordinated spatial position of the native caspofungin ligand was computationally transferred onto the modeled fungal structures. These aligned, multi-component complexes were subsequently exported as standalone PDB files and imported into the Schrödinger Suite (v2026.1) workspace for downstream simulations. Docking protocol validation by redocking the co-crystallized ligand yielded an RMSD of 1.2 Å, confirming reliable reproduction of the experimentally observed binding pose. Importantly, no explicit phospholipid bilayer was constructed or simulated in the docking workflow. The membrane shown in Figure 1 is a schematic representation provided solely to illustrate the transmembrane architecture of Fks1 and does not represent a computationally simulated membrane environment.

Before grid generation, the protein–ligand complexes were processed using the Protein Preparation Wizard module within the Schrödinger Suite to assign proper bond orders, add missing hydrogen atoms, optimize the hydrogen-bonding network, and alleviate potential steric strains through restrained minimization. The receptor grid generation was subsequently initiated using the Glide grid generation module. The position of the superimposed ligand was used to center the active-site bounding box, ensuring that the dimensions were sufficiently expanded to encompass both the large cyclic macrocycle and the long hydrophobic tail of the drugs. To accommodate local induced-fit adjustments during docking, conformational flexibility was introduced into the receptor grid by designating the side chains of Ser674 and Tyr675 in the representative *Trichophyton rubrum* Fks1 model as fully rotatable groups. Residue numbering in the other species-specific docking models was retained according to the corresponding modeled protein sequences. For species-specific interaction analyses, residue identities and numbering were reported directly from the corresponding modeled Fks1 sequence; therefore, numerical residue positions were not assumed to be identical across fungal species unless explicitly supported by the respective sequence/model.

Molecular docking simulations were subsequently executed utilizing the Glide SP-Peptide protocol. Given the rigid macrocyclic nature of the core hexapeptide ring and the highly flexible aliphatic/aromatic tails of the echinocandins, the search criteria were augmented by setting the docking thoroughness to a higher sampling effort (Thoroughness = 5.0). To bypass computational ring-strain divergence and retain biologically sound starting geometries, the sampling algorithm was configured to include and test the input ring conformation as a rigid template during the Monte Carlo conformational search. Docking simulations were carried out for both caspofungin and anidulafungin across all eight fungal targets, and the resulting poses were ranked based on the final Glide GScore (kcal/mol) values.

The docking analysis was designed as a comparative structural assessment of predicted ligand–target interaction patterns across dermatophyte species rather than as a quantitative determination of experimental binding affinity. Accordingly, docking scores were used only for relative comparison among the modeled ligand–protein complexes generated under the same computational protocol. The resulting poses and interaction maps were interpreted as structural predictions and were not considered experimental evidence of target engagement, thermodynamic binding affinity, or mechanism of antifungal action.

### 2.5. Statistical Analysis

Statistical analyses were performed using IBM SPSS Statistics [version 21], with data initially organized and maintained in Microsoft Excel. Normality testing indicated that the drug concentrations did not follow a normal distribution; therefore, nonparametric tests were employed. To compare caspofungin and anidulafungin with other drugs, the Kruskal–Wallis and Dunn’s tests were used, which demonstrated a statistically significant difference between these two drugs and the other drugs (*p* < 0.001). No formal correlation analysis was performed between species-level MEC values and docking scores. Susceptibility data were derived from individual clinical isolates and summarized by species, whereas docking generated a single model-derived score per species–drug combination; the two datasets were therefore not matched at the isolate level. Accordingly, the docking analysis was interpreted as a complementary structural assessment rather than a statistical predictor of antifungal susceptibility.

## 3. Results

### 3.1. Antifungal Susceptibility Testing

Table 1 summarizes the MEC_50_, MEC_90_, MEC ranges, geometric mean (GM) MEC values, and the mode MEC of anidulafungin and caspofungin compared with MIC values of standard antifungal agents, including terbinafine, itraconazole, voriconazole, and butenafine, against various dermatophyte species.

These findings demonstrate broad-spectrum in vitro activity of the evaluated echinocandins across the tested dermatophyte species, particularly against *T. mentagrophytes*, *T. tonsurans*, and *N. gypsea*, although modest species-dependent differences in MEC distributions were observed. Anidulafungin showed a MEC_50_ of 0.008 µg/mL and a GM MEC of 0.011 µg/mL, whereas caspofungin showed a MEC_50_ of 0.016 µg/mL and a GM MEC of 0.022 µg/mL. In the post hoc analysis, both echinocandins differed significantly from the comparator antifungal agents after adjustment for multiple comparisons. The adjusted *p-values* for anidulafungin and caspofungin versus itraconazole, voriconazole, terbinafine, and butenafine were all <0.001. Echinocandin activity remained stable among the 20 terbinafine-resistant *T. indotineae* isolates. Importantly, these isolates represented a previously molecularly characterized terbinafine-resistant subgroup, rather than a phenotypically defined resistance group alone. In our previous study of the *Trichophyton mentagrophytes*/*T. interdigitale* species complex from northern Iran, terbinafine resistance was investigated by sequencing the *SQLE* gene and was predominantly associated with the Phe^397^Leu substitution, either alone or in combination with Ala^448^Thr [23]. In the present study, these molecularly characterized isolates retained low echinocandin MECs despite elevated terbinafine MICs. Anidulafungin and caspofungin showed MEC_50_ values of 0.008 and 0.016 µg/mL, respectively, whereas the terbinafine MIC_50_ was 4 µg/mL. This observation provides phenotypic evidence that the terbinafine resistance background characterized by SQLE alterations was not accompanied by an apparent reduction in echinocandin activity. However, because the present study did not experimentally introduce or manipulate individual SQLE mutations, these findings do not establish a causal relationship between specific SQLE substitutions and echinocandin susceptibility. Rather, they demonstrate preserved echinocandin activity within a molecularly characterized terbinafine-resistant clinical isolate subset.

Across *T. rubrum*, *M. canis*, and *N. gypsea*, echinocandins demonstrated uniformly low MEC_50_ values (0.008–0.016 µg/mL), outperforming azoles and allylamines, even in species with generally favorable azole susceptibility. Although *T. violaceum* exhibited comparatively higher MEC values, echinocandins maintained activity comparable to or exceeding that of azoles.

### 3.2. Molecular Docking Analysis of Caspofungin and Anidulafungin

To evaluate the binding affinity and molecular interaction profiles of the clinically relevant echinocandins against β-(1,3)-D-glucan synthase, molecular docking simulations were systematically executed across eight fungal species. *Aspergillus fumigatus* was utilized as the primary reference strain for this comparative analysis, as it represents the established clinical benchmark against which echinocandin-target interactions are biologically validated in vivo. The optimized flexible-core, peptide-specific grid protocol successfully produced realistic, highly favorable negative docking energies (Table 2).

Molecular docking analysis of caspofungin binding to β-(1,3)-D-glucan synthase from *Trichophyton rubrum*, presented as a representative example of the seven dermatophyte species evaluated in this study. The right panel illustrates the overall three-dimensional structure of the AlphaFold3-predicted enzyme, whereas the left panel shows a magnified view of the predicted ligand interaction region, including the flexible residues Ser674 and Tyr675 surrounding the docked caspofungin molecule. The predicted docking score for this complex was −6.005 kcal/mol. The interaction map illustrates the predicted spatial arrangement of caspofungin within the modeled Fks1 interaction environment and provides a structural context for comparison with the corresponding complexes from the other fungal species (Figure 1).

**Figure 2:**
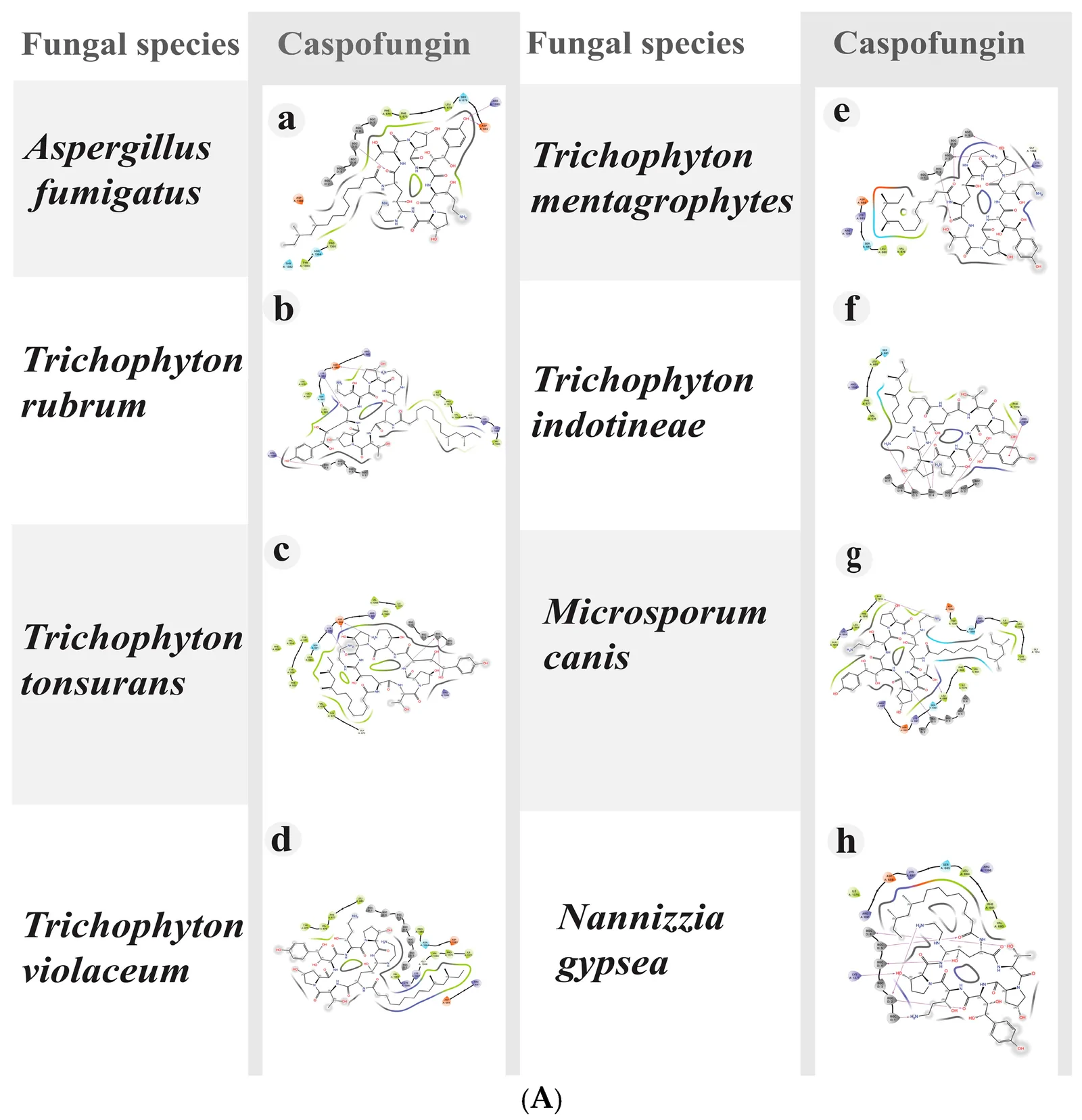

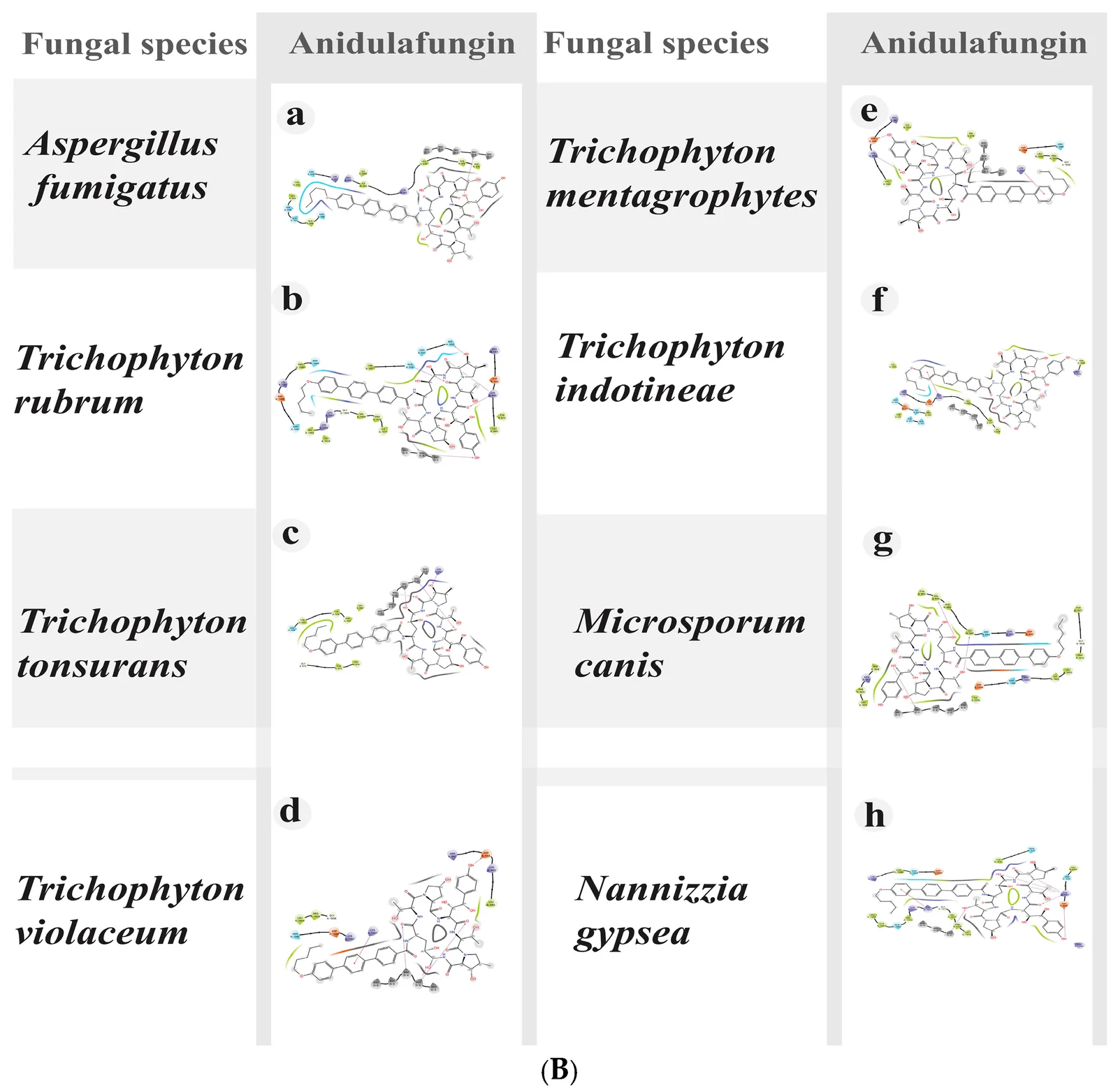
(**A**) Two-dimensional ligand–protein interaction diagrams of caspofungin with β-(1,3)-D-glucan synthase across eight fungal species. Panels (**a**–**h**) show the predicted interaction profiles of caspofungin with *Aspergillus fumigatus* (reference), *Trichophyton rubrum*, *T. tonsurans*, *T. violaceum*, *T. mentagrophytes*, *T. indotineae*, *Microsporum canis*, and *Nannizzia gypsea*, respectively. Dashed lines indicate hydrogen-bonding interactions, with corresponding distances shown in Å, whereas curved arcs represent hydrophobic contacts between caspofungin and surrounding protein residues. We highlight and label residues involved in polar interactions, and indicate solvent-exposed residues with pink/red circles. Ligand atoms are colored according to atom type (carbon, gray; oxygen, red; nitrogen, blue). The interaction diagrams were generated using Schrödinger Maestro (v2026.1) according to its standard two-dimensional interaction representation. (**B**) Two-dimensional ligand–protein interaction diagrams of anidulafungin with β-(1,3)-D-glucan synthase across eight fungal species. Panels (**a**–**h**) show the predicted interaction profiles of anidulafungin with *Aspergillus fumigatus* (reference), *Trichophyton rubrum*, *T. tonsurans*, *T. violaceum*, *T. mentagrophytes*, *T. indotineae*, *Microsporum canis*, and *Nannizzia gypsea*, respectively. Dashed lines indicate hydrogen-bonding interactions, with corresponding distances shown in Å, whereas curved arcs represent hydrophobic contacts between anidulafungin and surrounding protein residues. Residues involved in polar interactions are highlighted and labeled, and solvent-exposed residues are indicated by pink/red circles. Ligand atoms are colored according to atom type (carbon, gray; oxygen, red; nitrogen, blue). The interaction diagrams were generated using Schrödinger Maestro (v2026.1) according to its standard two-dimensional interaction representation.

Figure 2 present the two-dimensional ligand interaction plots, which illustrate the detailed binding modes and intermolecular contacts for each drug–target complex.

The reference docking scores for *A. fumigatus* were −6.138 kcal/mol for caspofungin and −5.067 kcal/mol for anidulafungin, providing reference values for comparison of the predicted binding of both echinocandins to β-(1,3)-D-glucan synthase (Table 2 and Figure 2A(a),B(a)). In comparison, several dermatophytes showed more favorable predicted binding scores than the *A. fumigatus* reference. For caspofungin, *Microsporum canis* showed the most favorable docking score (−9.061 kcal/mol), closely followed by *T. tonsurans* (−8.904 kcal/mol), whereas *T. violaceum* showed the least favorable score (−5.036 kcal/mol) (Table 2 and Figure 2A(c,d,g)). For anidulafungin, *N. gypsea* exhibited the most favorable docking score (−9.211 kcal/mol), followed by *M. canis* (−8.379 kcal/mol) and *T. rubrum* (−8.551 kcal/mol), while *T. mentagrophytes* showed the least favorable score (−5.129 kcal/mol) (Table 2 and Figure 2B(b,e,g,h)). These results indicate substantial species- and ligand-dependent variation in the predicted interaction of the echinocandins with β-(1,3)-D-glucan synthase.

Two-dimensional ligand–protein interaction plots indicate that the predicted interaction environments of caspofungin and anidulafungin differ across fungal species, particularly in the distribution of polar and nonpolar contacts. In the *A. fumigatus*–caspofungin complex, key residues involved are Ser679, Leu678, Phe674, Phe675, Arg1390, and Asp682, with Tyr1363, Asn1364, and Pro1365 in the vicinity. The interaction map highlights polar contacts between caspofungin and Arg1390 and Asp682, while Leu678, Phe674, and Phe675 are in a nonpolar environment (Figure 2A(a)). The overall docking score of −6.138 kcal/mol reflects the predicted interaction context rather than any single residue. In the *A. fumigatus*–anidulafungin complex, anidulafungin adopted an extended conformation, interacting with residues such as Tyr514, Leu509, and Ser508, while additional residues like Asn519 and Lys521 were nearby. The macrocyclic part of the ligand was associated with Lys681, Leu678, and Phe674. The two-dimensional interaction map revealed a mix of polar and nonpolar residues surrounding anidulafungin (Figure 2B(a)). The docking score of −5.067 kcal/mol indicates a less favorable interaction compared to several dermatophyte complexes, which had scores of −9.211 kcal/mol for *N. gypsea*, −8.551 kcal/mol for *T. rubrum*, and −8.379 kcal/mol for *M. canis*.

In *T. rubrum*, the predicted caspofungin interaction environment included Ser681, Leu680, Lys683, and Asp684, with Arg687 nearby. Additional residues, such as Tyr675 and Tyr507, were in the surrounding area, while Arg1392 was more distal (Figure 2A(b)). In this species-specific model, Ser681 and Tyr675 represent distinct residues within the predicted interaction environment, whereas Ser674 and Tyr675 were the flexible residues highlighted in the representative receptor grid setup. The docking score for the *T. rubrum*–caspofungin complex was −6.005 kcal/mol, slightly less favorable than the −6.138 kcal/mol score for the *A. fumigatus* reference complex. Conversely, the *T. rubrum*–anidulafungin complex had a more favorable docking score of −8.551 kcal/mol. Anidulafungin’s extended aromatic portion was surrounded by Tyr507, Tyr675, and Leu680, while its macrocyclic portion was near Lys683, Asp684, and Arg687. Other interacting residues included Gln510, Asn522, and Ser524 (Figure 2B(b)). The interaction map showed polar contacts with surrounding residues and hydrophobic contacts around the ligand. Overall, anidulafungin demonstrated a significantly better predicted docking score than caspofungin in *T. rubrum*, but the results do not confirm preferential binding or the underlying mechanism for the difference in affinity.

*Trichophyton tonsurans* displayed notable differences in the predicted docking scores for caspofungin and anidulafungin. Caspofungin had a docking score of −8.904 kcal/mol, with residues such as Ser681, Leu680, and Tyr675 in the species-specific interaction environment (Figure 2A(c)). In contrast, anidulafungin scored −5.550 kcal/mol, with its aromatic group interacting with residues like Tyr504 and Phe503 (Figure 2B(c)). Overall, caspofungin demonstrated a more favorable predicted docking score than anidulafungin (−8.904 vs. −5.550 kcal/mol), indicating relative docking energetics rather than experimental binding evidence.

*Trichophyton violaceum* exhibited the least favorable docking score for caspofungin at −5.036 kcal/mol, with key interactions involving Tyr675, Val676, Phe677, and Leu680 in a nonpolar environment, alongside residues like Asp684 and Arg687 in the ligand-associated region (Figure 2A(d)). In contrast, anidulafungin had a more favorable score of −6.022 kcal/mol, interacting with residues such as Arg687 and Lys683, and showing π-related contact with Lys1367 (Figure 2B(d)). The findings suggest that anidulafungin has a more favorable docking environment than caspofungin, yet do not confirm experimentally measured binding affinity or ligand selectivity.

*Trichophyton mentagrophytes* showed a more favorable predicted docking score for caspofungin than for anidulafungin (−7.445 vs. −5.129 kcal/mol; Table 2). The caspofungin interaction map included Ser681, Leu680, Val676, Lys683, and Asp684, together with distal residues including Lys1367 and Gly1368 (Figure 2A(e)). The map indicated polar contacts in the Lys683/Asp684 region and a surrounding nonpolar environment involving Leu680 and Val676. Anidulafungin showed a less favorable predicted score, with Lys683, Asp684, Arg687, and Leu680 among the residues in its interaction environment, together with additional distal residues (Figure 2B(e)). A π-associated contact between Lys1367 and the aromatic moiety of anidulafungin was also depicted. Overall, caspofungin showed more favorable predicted docking energetics in *T. mentagrophytes*; however, these results do not establish experimental binding affinity or preferential binding.

*Trichophyton indotineae* showed very similar predicted docking scores for caspofungin and anidulafungin (−6.702 and −6.600 kcal/mol, respectively; Table 2). In the caspofungin complex, the interaction map included Ser681, Leu680, Phe677, Val676, Arg1392, Arg1909, and Phe1910, with Leu680, Val676, and Phe677 positioned within the surrounding nonpolar environment (Figure 2A(f)). In contrast, the anidulafungin complex included Lys683, Asp684, Arg687, Leu680, and Val676, together with additional residues such as Asn522, Glu523, Ser524, Pro525, Tyr517, Thr511, Gln510, Phe677, Arg1909, and Phe1910 (Figure 2B(f)). The interaction map also depicts a π-associated contact involving the aromatic region of anidulafungin. Overall, the two ligands produced closely comparable predicted docking scores in *T. indotineae*, with no marked difference in the predicted docking energetics.

*Microsporum canis* showed favorable predicted docking scores for both echinocandins, with caspofungin yielding the most favorable score among all caspofungin complexes (−9.061 kcal/mol) and anidulafungin also showing a favorable score (−8.379 kcal/mol; Table 2). In the caspofungin complex, the interaction map included residues such as Phe685, Val684, Leu688, Ser689, Lys691, Asp692, and Arg695, together with additional distal residues (Figure 2A(g)). The anidulafungin complex showed an interaction environment including Tyr683, Val684, Phe685, Leu688, Ser689, Lys691, Asp692, and additional residues in proximal and distal regions (Figure 2B(g)). Both maps indicated combinations of polar and nonpolar interactions surrounding the ligands. Overall, both echinocandins showed favorable predicted docking scores in *M. canis*, with caspofungin exhibiting the more favorable predicted docking energetics (−9.061 vs. −8.379 kcal/mol); however, these computational results do not establish experimental binding affinity or preferential binding.

*Nannizzia gypsea* exhibited the most favorable predicted docking score for anidulafungin among all evaluated complexes (−9.211 kcal/mol), while caspofungin also showed a favorable score (−7.402 kcal/mol; Table 2). The caspofungin complex involved residues including Ser685, Leu684, Phe681, Val680, Lys687, Asp688, and Arg691, with additional distal residues such as Lys1371, Ile1375, and Arg1396 (Figure 2A(h)). The anidulafungin complex involved Tyr512, Gln515, Ser685, Leu684, Lys687, and Asp688, together with additional surrounding and distal residues, and showed a π-associated contact involving Lys1370 (Figure 2B(h)). Overall, anidulafungin showed a more favorable predicted docking score than caspofungin (−9.211 vs. −7.402 kcal/mol), although these computational results do not establish experimental binding affinity or a definitive mechanistic basis for this difference. Ser681 and Tyr1363 were observed in selected species-specific interaction environments, but neither residue was consistently represented as a conserved contact across all modeled dermatophytes.

Overall, the interaction maps indicated that the predicted docking interactions of caspofungin and anidulafungin with β-(1,3)-D-glucan synthase varied across both species and ligands, with no evidence of a single universally conserved interacting residue or residue pair. A recurrent central interaction region involving residues approximately within the 675–692 range was observed across several dermatophyte models; however, the specific residue identities and numbering varied among species. Leu, Val, Phe, and Tyr were frequently represented within the surrounding nonpolar environment, whereas Lys, Asp, Arg, Ser, Asn, and Gln were variably represented among the predicted polar contacts. Additional residues in more distal regions, particularly around positions 1365–1403, contributed to the broader interaction environment in selected complexes. Thus, the docking results identify a recurrent spatial interaction environment rather than a universally conserved residue-level interaction pattern. Although these species- and ligand-dependent interaction patterns coincided with differences in the predicted docking scores, the interaction maps alone do not establish the energetic contribution of individual contacts or a definitive mechanistic basis for the calculated scores.

Importantly, the docking results should be interpreted as computational predictions of relative docking favorability rather than direct measurements of experimental binding affinity, thermodynamic stability, or antifungal susceptibility. The data do not by themselves establish ligand-induced conformational dynamics or demonstrate that any particular residue is universally responsible for echinocandin recognition. Nevertheless, the species-specific interaction patterns were associated with differences in predicted docking scores. Caspofungin showed its most favorable predicted scores against *M. canis* (−9.061 kcal/mol) and *T. tonsurans* (−8.904 kcal/mol), whereas anidulafungin showed its most favorable scores against *N. gypsea* (−9.211 kcal/mol), *T. rubrum* (−8.551 kcal/mol), and *M. canis* (−8.379 kcal/mol) (Table 2).

## 4. Discussion

Antifungal susceptibility testing of 105 clinical dermatophyte isolates demonstrated that anidulafungin and caspofungin had superior in vitro potency compared to azole and allylamine drugs. Both echinocandins showed low MEC values, with significant differences relative to other antifungals (*p* < 0.001). This highlights their strong activity, especially against *T. mentagrophytes*, *T. tonsurans*, and *N. gypsea*. In the broader context of the increasing antimicrobial resistance burden, recent advances in synthetic biology have emphasized complementary approaches to antifungal discovery, including the identification of novel fungal targets and the development of innovative antifungal delivery strategies [25].

The consistent superiority of anidulafungin and caspofungin aligns with multiple international and national studies. Bao et al. (2013) demonstrated that micafungin exhibited the lowest MEC_90_ among echinocandins and highlighted its substantial in vitro activity against dermatophytes [18]. Similar findings by Badali (2015) identified anidulafungin as the most potent agent among nine antifungals tested [16]. Studies by Su et al. (2023) confirmed the remarkably low MECs of anidulafungin and micafungin against *T. rubrum* [19]. Importantly, because no CLSI clinical breakpoints or ECVs are currently available for echinocandin susceptibility testing of dermatophytes, the present findings should be interpreted as comparative in vitro susceptibility data rather than evidence of clinical susceptibility or resistance. The global emergence of terbinafine-resistant *T. indotineae*, as highlighted by McTaggart (2025), further underscores the clinical need for alternatives such as echinocandins [26]. Recent studies by Xie et al. (2025) consistently reported the evaluation of 16 antifungal agents against *T. indotineae*, in this research, echinocandins showed potent in vitro activity against these resistant species [20]. Other studies, including those by Badali (2015), Baghi (2016), and Haghani (2019), similarly reported strong in vitro activity of anidulafungin and caspofungin, alongside newer azoles such as luliconazole [16,17,27,28].

Comparative analyses revealed significant increases in the median MIC values of azole and butenafine in terbinafine-resistant isolates (*p* < 0.001). The consistent superiority of anidulafungin, reflected in the lowest MEC_50_, MEC_90_, and GM values across all species, showed that it had the lowest in vitro MEC values among the agents evaluated.

The present study extends previous observations by characterizing echinocandin susceptibility in a molecularly contextualized terbinafine-resistant *T. indotineae* subgroup within a broader, multi-species clinical dermatophyte collection [20]. These observations align with mechanistic insights by Martinez-Rossi (2018), who emphasized the independence of the β-(1,3)-D-glucan synthase pathway targeted by echinocandins from the SQLE-dependent pathway involved in terbinafine resistance [29]. The preserved echinocandin activity observed in the terbinafine-resistant *T. indotineae* subgroup is biologically consistent with the fact that terbinafine and echinocandins act on distinct antifungal pathways. However, because the present study was not designed to experimentally manipulate individual SQLE mutations or measure their direct effects on Fks1, these findings should be interpreted as phenotypic evidence of preserved echinocandin susceptibility in a molecularly characterized terbinafine resistance background rather than as proof that specific SQLE substitutions cannot affect echinocandin susceptibility [19].

The novelty of this study does not reside in identifying Fks1 as an echinocandin target, but rather in integrating species-resolved structural predictions with standardized susceptibility data obtained from a clinically relevant multi-species dermatophyte collection that included a molecularly characterized terbinafine-resistant *T. indotineae* subgroup. The analysis included seven dermatophyte species and permitted direct comparison of predicted caspofungin and anidulafungin interaction environments under a common computational framework. Importantly, this structural analysis was performed alongside standardized susceptibility testing and included *T. indotineae* isolates with a previously molecularly characterized SQLE-associated terbinafine resistance background [23]. The combined dataset provides a comparative framework for examining whether the phenotypic preservation of echinocandin activity in terbinafine-resistant isolates is accompanied by broadly preserved predicted interaction with the modeled Fks1 target. Although these observations do not establish causality or experimental target engagement, they provide a hypothesis-generating basis for subsequent biochemical, mutational, and structural investigations of echinocandin activity against dermatophytes.

To further contextualize the observed in vitro activity, molecular docking simulations were performed to characterize the predicted interaction environments of anidulafungin and caspofungin within fungal β-(1,3)-D-glucan synthase (Fks1). The docking analysis was intended as a complementary structural assessment and was not designed to establish experimental binding affinity, target engagement, antifungal efficacy, or a statistical relationship between docking scores and MEC values. Both echinocandins produced negative docking scores against the *A. fumigatus* reference model (caspofungin: −6.138 kcal/mol; anidulafungin: −5.067 kcal/mol), providing a comparative structural benchmark rather than a quantitative measure of binding. Across dermatophyte models, docking scores varied according to both fungal species and ligand. Caspofungin showed its most favorable predicted scores against *M. canis* (−9.061 kcal/mol) and *T. tonsurans* (−8.904 kcal/mol), whereas anidulafungin showed its most favorable scores against *N. gypsea* (−9.211 kcal/mol), *T. rubrum* (−8.551 kcal/mol), and *M. canis* (−8.379 kcal/mol) (Table 2). Similarly, both agents produced comparable docking scores against terbinafine-resistant *T. indotineae* (−6.702 and −6.600 kcal/mol, respectively), consistent with the preserved echinocandin activity observed phenotypically in this molecularly characterized resistant subgroup.

Analysis of the predicted interaction maps indicated that both echinocandins occupied corresponding regions of the modeled Fks1 proteins, although the surrounding interaction environments varied among species. A recurrent interaction region encompassing residues approximately within the 675–692 interval was observed across several dermatophyte models; however, the specific residue identities and numbering differed. Rather than a universally conserved residue pair, the models demonstrated variable combinations of polar residues (Ser, Lys, Asp, Arg, Asn, and Gln) and nonpolar residues (Leu, Val, Phe, and Tyr), with additional contributions from more distal residues within the approximately 1365–1403 region in selected species. These findings support a conserved interaction region with species-dependent residue composition rather than a universally conserved hydrogen-bonding network.

This interpretation is consistent with recent structural studies demonstrating that echinocandin recognition occurs within a complex membrane-associated Fks1 environment and involves both direct ligand interactions and indirect effects mediated through nascent glucan and surrounding structural elements [21,22,30]. Notably, the recent cryo-EM structure of caspofungin-bound *Saccharomyces cerevisiae* Fks1 showed that resistance-associated substitutions can influence drug recognition through both steric and allosteric mechanisms within a ternary Fks1-glucan-drug complex [21]. Although favorable MEC values and predicted docking interactions were observed concurrently, the present data do not establish a causal or statistical relationship between specific residues, docking scores, and antifungal susceptibility. Establishing such relationships will require experimentally resolved dermatophyte Fks1 structures, site-directed mutagenesis, biochemical target-engagement studies, and structure-activity analyses [21]. Nevertheless, when considered together with the susceptibility data, the docking results provide a biologically plausible structural framework supporting the broad in vitro activity of echinocandins, including against terbinafine-resistant *T. indotineae*, while highlighting interspecies variation in predicted ligand–target interactions [19,20,21,22,30,31].

Given the increasing prevalence of terbinafine-resistant *T. indotineae* and the therapeutic challenges associated with recalcitrant dermatophytosis [32], the present findings identify echinocandins as candidates for further preclinical investigation rather than established clinical alternatives. Although anidulafungin and caspofungin demonstrated consistently low MEC values in vitro, these results should not be interpreted as evidence of clinical efficacy or as a basis for recommending either agent for routine treatment of dermatophytosis. Translation of in vitro activity into clinical benefit requires adequate drug exposure at the site of infection, appropriate PK/PD target attainment, favorable tissue distribution, and demonstrable clinical and mycological efficacy, a distinction particularly relevant because both agents are administered intravenously and are primarily indicated for invasive fungal infections rather than superficial dermatophytosis [30]. Recent evidence similarly indicates favorable in vitro activity of echinocandins against *T. indotineae*, while their therapeutic potential remains uncertain because of PK/PD limitations [20]. Therefore, the low MEC values observed here should be regarded as a preclinical signal warranting further investigation rather than as a surrogate for clinical effectiveness.

Ultimately, clinical adoption would require structured treatment protocols and phase II/III randomized trials to establish efficacy, safety, and optimal dosing, together with formal pharmacoeconomic evaluation of cost-effectiveness [33,34]. Only after adequate preclinical and clinical validation can the therapeutic role of echinocandins in dermatophytosis be determined.

Importantly, the present study has an inherent translational limitation because no animal model of dermatophytosis was included. An additional limitation is that the present study did not determine whether the experimentally observed MECs correspond to drug exposures achievable within infected human skin, hair, or nail tissues. This distinction is particularly important for dermatophytosis because the anatomical site of infection and the degree of keratinization can substantially influence antifungal exposure and therapeutic response. Although the low MECs observed for anidulafungin and caspofungin provide reproducible in vitro evidence of activity, susceptibility measurements cannot account for host–pathogen interactions, tissue penetration, drug exposure at the site of infection, PK/PD relationships, or the influence of the cutaneous microenvironment on therapeutic response. Therefore, the present findings should be regarded as a preclinical in vitro and computational basis for further investigation rather than evidence of in vivo or clinical efficacy. In this context, Hager et al. evaluated rezafungin versus terbinafine for treating dermatophytosis caused by *T. mentagrophytes* in a guinea pig model. Male albino guinea pigs were randomly assigned to receive rezafungin (subcutaneous; 10, 20, or 40 mg/kg), terbinafine (oral; 10 mg/kg), or a vehicle control, administered on days 1 and 8. Efficacy was assessed through clinical and mycological evaluations on day 12 post-challenge. The vehicle control group exhibited expected hair loss and skin lesions, while rezafungin demonstrated efficacy of 90.5%, 94.2%, and 98.4% for the respective doses. Terbinafine achieved 76.8% efficacy, with all treatment groups significantly outperforming the vehicle control (*p* < 0.001). Rezafungin also showed significant superiority over terbinafine (*p* ≤ 0.001), supporting further clinical evaluation for treating dermatophyte infections [35].

Despite the potent in vitro activity of the current echinocandins (anidulafungin and caspofungin), their formulations are unsuitable for topical use due to their structural and physicochemical properties, including: high molecular weight (1093.31 g/mol), limiting penetration through keratinized tissue; amphipathic, cyclic peptide structure hindering trans-epidermal absorption; and instability in the cutaneous microenvironment (acidic pH and enzymatic degradation) [36,37,38]. In recent years, formulations combining natural and synthetic polymers have been developed to create multifunctional gels that enhance the penetration of macromolecules, such as echinocandins.

Moreover, advanced nanotechnology-based delivery systems may overcome these barriers. Potential strategies include nanoemulsions, nanogels, and nano-creams for enhanced dispersion and prolonged skin residence; lipid-based carriers (liposomes, SLN, NLC) enabling improved epidermal penetration and controlled release; transfersomes and ethosomes designed to bypass the stratum corneum; polymeric nanoparticles or covalent prodrug systems; and microneedles and hydrogel-microneedle systems enabling direct delivery into deeper or follicular tissues [39,40,41,42].

Liposomal echinocandin formulations have recently emerged as a promising approach to enhance antifungal efficacy. Due to their large size, high polarity, and limited tissue penetration, echinocandins are currently restricted to intravenous use. However, liposomal encapsulation may circumvent these limitations [43]. Vera-González et al. (2020) demonstrated that liposomal anidulafungin markedly improved stability, solubility, penetration, and activity against both planktonic and biofilm forms of *Candida albicans*, outperforming the free drug [43]. They developed liposomal nanoparticles incorporating anidulafungin to enhance its solubility and target fungal cells. These nanoparticles, around 100 nm in size, effectively inhibited the growth of planktonic *C. albicans* and disrupted preformed biofilms, reducing fungal burden by up to 99%, significantly more than the free drug. In vivo tests on *C. albicans*-infected *Galleria mellonella* wax moth larvae showed increased survival rates of 33% to 67% with the liposomal formulations, compared to 25% with the free drug. Importantly, neither the liposomal formulations nor the free anidulafungin caused red blood cell lysis. Overall, these liposome formulations improved anidulafungin’s solubility and efficacy against *C. albicans* [43]. Pérez-González et al. (2023) reported that penetration-enhanced caspofungin gels significantly increased dermal absorption and antifungal efficacy in vitro and ex vivo, suggesting a potential transition from injectable to topical formulations and that dermal treatment with caspofungin may serve as an effective therapy for cutaneous candidiasis in patients who are resistant or intolerant to traditional antifungal agents [36]. Currently, there are no studies specifically evaluating liposomal echinocandins against dermatophytes, highlighting a significant gap that needs exploration due to the consistent in vitro activity shown in this study.

Additional advanced delivery systems have shown promise in non-dermatophyte fungal infections. Zhang et al. (2021) developed human albumin nanoparticles loaded with anidulafungin, achieving markedly improved solubility, protection from enzymatic degradation, reduced MICs, and enhanced survival in murine models of candidiasis, with up to a 30-fold increase in potency versus the free drug [44]. Macwan et al. focused on developing a caspofungin liposome aimed at improving ocular delivery by enhancing corneal penetration. A Quality by Design approach was employed to identify the critical factors that affect the final liposomal formulation. The liposome was prepared using a thin-film hydration method and characterized to assess its physicochemical properties, irritation potential, and corneal uptake. The optimized formulation demonstrated no signs of irritation and showed significant corneal permeation. This liposomal formulation enhanced the permeability of caspofungin, potentially improving its efficacy in treating conditions such as fungal keratitis [45].

## 5. Conclusions

Collectively, the present findings provide additional comparative evidence of reproducible in vitro activity of anidulafungin and caspofungin against clinically relevant dermatophytes, including terbinafine-resistant *T. indotineae*. The preserved echinocandin MECs observed in the molecularly characterized terbinafine-resistant subgroup provide phenotypic evidence of maintained echinocandin activity despite elevated terbinafine MICs. Molecular docking provides complementary, species-resolved structural context for these susceptibility profiles but does not establish experimental target engagement, binding affinity, or therapeutic efficacy. The absence of an animal infection model represents an important translational limitation of the present study, and the current findings should therefore not be interpreted as evidence of in vivo or clinical efficacy. Future investigations will incorporate validated animal models of dermatophytosis, together with PK/PD analyses, tissue-exposure studies, formulation optimization, biochemical target-engagement assays, site-directed mutagenesis, and experimentally resolved dermatophyte Fks1 structures, to determine whether the favorable in vitro activity observed here can be translated into meaningful in vivo antifungal effects.

## Figures and Tables

**Table 1:** In vitro susceptibility of 105 different species of dermatophytes to echinocandins and 4 routine antifungal agents.

Species	Antifungal Agent	MIC or MEC (μg/mL)				
		MIC or MEC Range	GM	MIC_50_ or MEC_50_	MIC_90_ or MEC_90_	Mode
*Trichophyton indotineae* (*n* = 20)	ITZ	0.016–16	0.139	0.125	0.5	0.032
	VRC	0.016–1	0.075	0.063	0.25	0.125
	TER	4–16	6.276	4	16	4
	BTN	4–16	6.727	4	16	4
	ANF	0.008–0.063	0.010	0.008	0.032	0.008
	CAS	0.008–0.063	0.017	0.016	0.063	0.016
*Trichophyton mentagrophytes* (*n* = 20)	ITZ	0.016–2	0.067	0.063	0.25	0.063
	VRC	0.016–0.5	0.036	0.032	0.125	0.032
	TER	0.016–4	0.121	0.125	0.5	0.25
	BTN	0.016–16	0.197	0.125	0.5	0.125
	ANF	0.008–0.032	0.009	0.008	0.008	0.008
	CAS	0.008–0.032	0.013	0.016	0.016	0.016
*Trichophyton tonsurans* (*n* = 20)	ITZ	0.016–0.125	0.035	0.032	0.063	0.032
	VRC	0.032–0.063	0.035	0.032	0.063	0.032
	TER	0.016–4	0.150	0.063	2	0.063
	BTN	0.032–4	0.191	0.094	2	0.032
	ANF	0.008–0.032	0.010	0.008	0.032	0.008
	CAS	0.008–0.063	0.019	0.016	0.032	0.032
*Trichophyton rubrum* (*n* = 10)	ITZ	0.016–0.5	0.059	0.032	0.25	0.016
	VRC	0.016–0.25	0.039	0.032	0.063	0.032
	TER	0.016–0.032	0.024	0.016	0.032	0.016
	BTN	0.032–0.125	0.036	0.032	0.125	0.032
	ANF	0.008–0.125	0.022	0.016	0.125	0.016
	CAS	0.016–0.125	0.036	0.016	0.125	0.016
*Trichophyton violaceum* (*n* = 5)	ITZ	0.016–0.5	0.165	0.25	0.5	0.5
	VRC	0.016–0.125	0.047	0.063	0.125	0.063
	TER	0.016–0.032	0.024	0.032	0.032	0.032
	BTN	0.016–0.063	0.027	0.032	0.063	0.032
	ANF	0.016–0.125	0.036	0.032	0.125	0.032
	CAS	0.032–0.5	0.109	0.125	0.5	0.125
*Microsporum canis* (*n* = 20)	ITZ	0.016–0.032	0.017	0.016	0.032	0.016
	VRC	0.016–0.32	0.016	0.016	0.016	0.016
	TER	0.016–0.063	0.025	0.016	0.063	0.016
	BTN	0.032–0.125	0.037	0.032	0.063	0.032
	ANF	0.008–0.016	0.008	0.008	0.016	0.008
	CAS	0.008–0.063	0.030	0.032	0.063	0.063
*Nannizzia gypsea* (*n* = 10)	ITZ	0.016–2	0.039	0.032	0.032	0.032
	VRC	0.016–0.5	0.025	0.016	0.032	0.016
	TER	0.016–1	0.0391	0.032	0.063	0.016
	BTN	0.032–1	0.055	0.032	0.125	0.032
	ANF	0.008–0.032	0.010	0.008	0.016	0.008
	CAS	0.008–0.063	0.018	0.016	0.032	0.008
All spp. (*n* = 105)	ITZ	0.016–16	0.052	0.032	0.032	0.25
	VRC	0.016–1	0.035	0.032	0.032	0.125
	TER	0.016–16	0.141	0.063	0.063	4
	BTN	0.016–16	0.192	0.063	0.125	8
	ANF	0.008–0.125	0.011	0.008	0.016	0.032
	CAS	0.008–0.5	0.022	0.016	0.032	0.063

Abbreviations: MEC, minimum effective concentration; GM, geometric mean; ITZ, itraconazole; VRC, voriconazole; TER, terbinafine; BTN, butenafine; ANF, anidulafungin; CAS, caspofungin. Mode: It refers to the most frequently observed MIC or MEC value among the tested isolates for each species–drug combination, representing the most typical concentration needed to inhibit the respective isolate population.

**Table 2:** Docking scores (kcal/mol) of caspofungin and anidulafungin against β-(1,3)-D-glucan synthase across eight fungal species.

Organisms	Drug	Docking Scores (kcal/mol)
*Trichophyton rubrum*	Caspofungin	−6.005
	Anidulafungin	−8.551
*Nannizzia gypsea*	Caspofungin	−7.402
	Anidulafungin	−9.211
*Microsporum canis*	Caspofungin	−9.061
	Anidulafungin	−8.379
*Trichophyton tonsurans*	Caspofungin	−8.904
	Anidulafungin	−5.550
*Trichophyton violaceum*	Caspofungin	−5.036
	Anidulafungin	−6.022
*Trichophyton mentagrophytes*	Caspofungin	−7.445
	Anidulafungin	−5.129
*Trichophyton indotineae*	Caspofungin	−6.702
	Anidulafungin	−6.600
*Aspergillus fumigatus* (Reference)	Caspofungin	−6.138
	Anidulafungin	−5.067

## Data Availability

The datasets generated and/or analyzed during the current study are available from the corresponding author upon reasonable request. Data are stored in electronic format using Microsoft Excel and IBM SPSS Statistics [version 21] software.

## Abbreviations

The following abbreviations are used in this manuscript:

CLSI	Clinical and Laboratory Standards Institute
MEC	Minimum effective concentration
MIC	Minimum inhibitory concentrations
GM	Geometric Mean
MOPS	N-morpholine propane sulfonic acid
DMSO	Dimethyl sulphoxide
SQLE	Squalene Epoxidase
ITZ	Itraconazole
VRC	Voriconazole
TER	Terbinafine
BTN	Butenafine
ANF	Anidulafungin
CAS	Caspofungin
